# In Vitro conservation and genetic diversity analysis of rare species *Ribes janczewskii*

**DOI:** 10.1038/s41598-024-82320-y

**Published:** 2024-12-28

**Authors:** Aidana Nurtaza, Damira Dyussembekova, Symbat Islamova, Indira Samatova, Zhanargul Zhanybekova, Alima Umirzakova, Gulmira Magzumova, Anna Muranets, Almagul Kakimzhanova

**Affiliations:** 1 National Center for Biotechnology, 13/5, Korgalzhyn Road, 010000 Astana, Kazakhstan; 2 Department of Science, Information and Monitoring, Sairam-Ugam State National Nature Park, 24 1, Ilyaev Street, 160011 Shymkent, Kazakhstan

**Keywords:** *Ribes janczewskii*, Micropropagation, Slow-growth storage, DNA barcoding, iPBS, Plant biotechnology, Plant genetics, Plant molecular biology

## Abstract

*Ribes janczewskii* is a rare and valuable plant known for its resistance to spring frosts, pests, and diseases. It is used in hybridization to develop resistant currant varieties but is on the verge of extinction, listed in Kazakhstan Red Book. This study developed a micropropagation and slow-growth storage protocol for conservation. Genotypes were identified through DNA barcode analysis (*rbcL*, ITS, and *matK*) and sequences uploaded to the National Center for Biotechnology Information database. Genetic diversity was assessed using iPBS primers, generating 98 fragments with 88–94% polymorphic bands. Biochemical analysis of fruits showed vitamin C content from 4.64 to 5.61 mg/100 g, vitamin E from 2.26 to 3.16 mg/100 g, vitamin B5 from 3.18 to 4.93 mg/100 g, and quercetin up to 12.5 mg/100 g. Micropropagation stages were optimized with 12% hydrogen peroxide for surface sterilization, achieving up to 73.3% explant viability. Effective hormonal combinations for in vitro culture included WPM with BAP 0.2 mg L^−1^ and GA 0.5 mg L^−1^, and for propagation, BAP 0.25 mg L^−1^, GA 0.5 mg L^−1^, and IBA 0.5 mg L^−1^. Mannitol (20 g L^−1^) was used for slow-growth storage, keeping explants viable for 4 months without re-cultivation.

**Supplementary Information:**

The online version contains supplementary material available at 10.1038/s41598-024-82320-y.

## Introduction

Conservation of biodiversity is of global importance for sustaining ecosystems and preserving natural resources. Land-use change and urban expansion have significantly impacted natural ecosystems^1^. The current rate of plant extinction is concerning, with estimates suggesting the loss of one species per day^2^. This trend, if it continues, could result in the extinction of a substantial number of plant species over the coming decades. Recent studies indicate that a significant proportion of the world’s plant species are at risk of becoming threatened^3,4^, and 50% of the world’s plants are endemic^5^. Kazakhstan occupies a leading place among the Central Asian states in terms of plant diversity. Approximately 5700 vascular plants grow in Kazakhstan, of which approximately 14% are endemic, and many are endangered^6^. *Ribes janczewskii* Pojark is an example of a species that is negatively affected.

*R. janczewskii* is a rare species and a valuable selection stock and food plant. It is a shrub plant, 100–150 cm high, with erect branches, glabrous or sparse villous, golden, later becoming off-yellow sprouts. The leaves are thin and brilliant, with yellow odorous ribs in the lower part, deeply cordate, and serrated along the edge. The racemes comprise 5–10 flowers. The flowers are pale yellow, and the hypanthium is campanulate and not tightened on the tip. The sepals are obtuse. The petals are broad and ovoid. The berries are spherical, up to 13 mm long, black, and fragrant. Florification of the plant occurs in July, and fruiting occurs in August. It occurs as solitary plants or in small communities on stony slopes of median mountain zones, placers, and river valleys in the Ketmen and Terskey ranges in Trans-Ili Alatau^7^.

Currently, *R. janczewskii* is on the verge of extinction and is listed in the Red Book of Kazakhstan. *R. janczewskii* has valuable traits such as resistance to spring frosts due to late flowering and high resistance to pests and diseases (powdery mildew and septoria leaf spot). It is used for hybridisation to obtain resistant forms of currants^8^. This species is important for evolution, as it provides all user groups, including breeders and researchers, with invaluable genetic material^9^. Blackcurrant fruits are rich in ascorbic acid, polyphenols, and anthocyanins^10^. However, it is important to note that there are no publications on the composition of biologically active substances in *R. janczewskii* fruits.

However, this species forms small populations and grows in the gorges and valleys of rivers in the mountains of Central Asia^11^. Thus, the limited amount of plant material and inaccessibility of individuals make it difficult to use traditional methods of propagation and conservation of the species. Biotechnological methods are effective in solving this problem. In vitro cultivation and slow-growth storage are valuable techniques for the large-scale propagation, conservation, and reintroduction of endangered plant species. Effective protocols have been developed for conserving many tree crops, including strategically important crops such as currants, which are maintained in vitro through the slow-growth storage of shoots^12–14^. Over the past decades, significant progress has been made in developing methods for the slow- and long-term storage of plant species^15,16^. Researchers use micropropagation for large-scale production and short-term preservation of cultivated currant species of the *Ribes* germplasm^17^. However, research related to the development of biotechnology for the wild, endangered species *R. janczewskii* has not yet been published.

Thus, the aim of this study was to examine wild specimens of *R. janczewskii* in Kazakhstan. Molecular genetic analysis of individuals from different specimens. Developing a protocol for micropropagation and slow-growth storage of *R. janczewskii*. To the best of our knowledge, this is the first study on the biochemical composition of fruits with the aim of creating a collection of in vitro cultures for the conservation of the endangered species *R. janczewskii*.

## Results

### DNA barcoding

Six samples were selected for molecular genetic identification. This allowed us to conclude that this species is significantly represented in the location under consideration and emphasises the importance of studying its genetic characteristics.

Studies have confirmed the effectiveness of the markers used in the amplification of products, making it possible to successfully obtain nucleotide sequences for further analysis. The minimum and maximum length ranges (in base pairs, bp) for the ITS2, *matK*, and *rbcL* regions were 496–683, 432–779, and 425–569, respectively. The average lengths of each region were 562.3, 663.3 and 523.2 bp, respectively.

The GC content (%) for the ITS2, *matK*, and *rbcL* regions ranged from 55.2 to 57.2%, 35.8 to 37.3%, and 43.8 to 45.4%, respectively. The mean GC values were 56.37%, 36.07%, and 44.28%, respectively. These GC content data provide additional information regarding the structural features of the studied regions of *R. janczewskii*.

All nucleotide sequences were manually edited using the SeqMan software to eliminate possible errors and improve the accuracy of the data. We used the BLAST method in the NCBI database to analyse the similarity of the obtained sequences, enabling us to assess the degree of similarity with other known sequences. The results showed a high level of similarity, ranging from 98 to 100%, for all three markers. The sequencing results were also uploaded to the international NCBI database, where each of the six studied specimens was assigned accession numbers for the ITS2, *matK*, and *rbcL* primers (Table 1).

**Table 1:** Information of DNA barcoding result.

Sample ID	ITS		matK		rbcL	
	GC content	Accession number	GC content	Accession number	GC content	Accession number
1	56.3	PP702917	35.8	PP708896	44.9	PP493202
2	55.2	PP702918	36.0	PP708897	43.8	PP493204
3	57.2	PP464114	35.8	PP708898	43.8	PP493205
4	55.4	PP702919	36.0	PP708899	43.9	PP493206
5	56.9	PP464115	35.5	PP708900	45.4	PP493207
6	57.2	PP709330	37.3	PP708901	43.9	PP708902

#### Assessment of genetic diversity

Primers were selected based on their ability to produce clear and reproducible PCR amplicons, which are crucial for accurate genetic analysis. Electrophoretic separation of the PCR products resulted in amplicons ranging in size from 650 to 4000 base pairs (bp). Electropherograms of the amplification results provided insights into the diversity of the samples. They revealed the presence of both common amplicons, consistent across all specimens, and unique amplicons specific to each sample. This diversity underscores the genetic variation among the studied specimens (Supplementary file 1).

To ensure the reliability of the analysis, each DNA sample was subjected to testing at least three times. This rigorous approach confirmed the reproducibility of the results and enabled the identification of specific iPBS profiles, which are distinctively associated with each specimen of *R. janczewskii*. Although fourteen primers were initially evaluated in the study (Table 5), only these four (2224, 2228, 2229, and 2393) produced informative results across all six specimens.

The amplification profiles generated using iPBS primers were further evaluated using informative parameters such as polymorphic information content (PIC). These parameters served as indicators of the ability of the primers to detect genetic variation among *R. janczewskii* specimens. Of the primers investigated, primers 2224, 2228, 2229, and 2393 were highly informative.

**Fig. 1:**
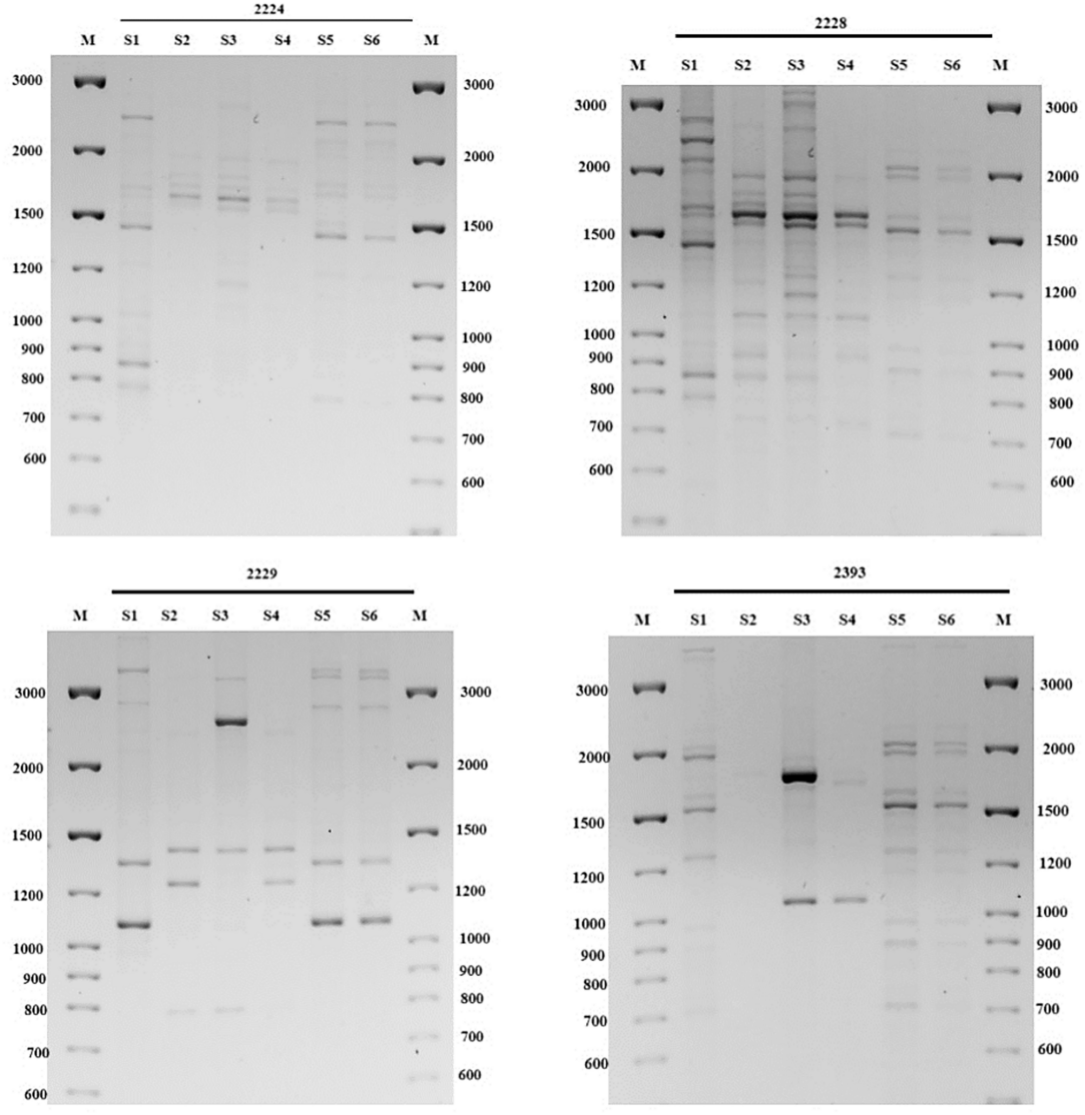
Electrophoretic pattern of *R. janczewskii* fragments using iPBS primer 2224, 2228, 2229 and 2393.

A total of 98 fragments were generated in the present study (Fig. 1). Primer 2229 produced the lowest number of amplified fragments (18), whereas primer 2228 yielded the highest number (34). Primers 2224 and 2393 amplified 23 fragments (Fig. 1). The proportion of polymorphic bands varied across the primers, with primer 2229 exhibiting the highest proportion (94%) and primer 2228 the lowest (88%). The polymorphism index (PIC) further reflected the discriminatory power of the primers, with primer 2229 having the highest PIC (0.41) and primer 2228 having the lowest (0.35), indicating their effectiveness in capturing genetic diversity within *R. janczewskii* specimens.

### Biochemical composition of berries

The biochemical composition of selected specimens was studied to determine and compare the nutritional value and beneficial properties of *R. janczewskii* fruits. A study on *R. janczewskii* fruits revealed significant amounts of biologically active substances that are important in maintaining human health. Analysis of vitamin content showed that the level of vitamin C ranged from 4.64 ± 0.46 to 5.61 ± 0.56 mg/100 g wet weight, vitamin E from 2.26 ± 0.23 to 3.16 ± 0.32 mg/100 g wet weight, and vitamin B5 from 3.18 ± 0.32 to 4.93 ± 0.49 mg/100 g wet weight.

The content of the flavonoid quercetin ranged from 12.2 ± 2.44 to 12.5 ± 2.5 mg/100 g fresh weight, highlighting the high nutritional value of these fruits. No statistically significant differences were found in the content of biologically active substances between different specimens of *R. janczewskii*, indicating the stability of the nutritional composition regardless of the place of origin.

These results highlighted the importance of *R. janczewskii* as a valuable source of vitamins and other biologically active compounds that promote health and strengthen the immune response. In this regard, the use of the rare species *R. janczewskii* for crossing cultivated varieties of black currant is relevant.

### Sterilisation and establishment in vitro culture

To optimize the conditions of the micropropagation stages, specimens 2 was used. Surface sterilisation of explants is the primary task in establishing in vitro cultures. This study examined the effectiveness of different concentrations of hydrogen peroxide for the sterilisation of the axillary buds of *R. janczewskii*.

**Fig. 2:**
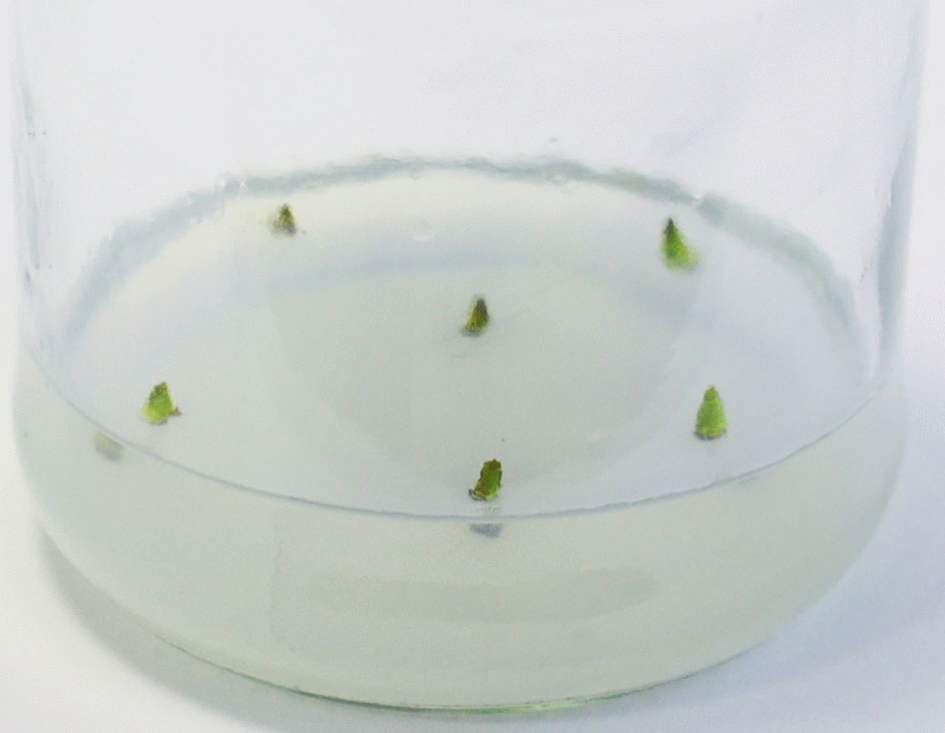
Sterile and viable explants of *R. janczewskii.*

As the results showed, high contamination was observed in treatment I. Infection with pathogenic microflora was observed in 13 out of 15 explants, which amounted to 86.7%. Explant viability was 13.3%. An increase in the concentration of hydrogen peroxide to 24% (treatment III) led to necrosis in up to 66.7% of the explants. Ten explants experienced burns, and only five explants remained viable. The mildest but most effective method of sterilisation among those considered was a solution of 12% hydrogen peroxide (treatment II); 11 explants were sterile and retained their viability (73.3%) (Fig. 2). Growth of pathogenic microflora was observed in only three explants; one explant experienced a burn.

Thus, a 12% hydrogen peroxide solution was effective for surface sterilisation of the axillary buds of *R. janczewskii*. A high degree of explant viability (up to 73.3%) and a low percentage of contamination were obtained.

### Regeneration of the main shoot

Various growth regulators are used for micropropagation and the choice of optimal propagation protocol depends on the plant species. For example, for the propagation of certain species of currant at the stage of regeneration of the main shoot, the authors recommend using BAP 2.0 mg L^−1^ and IBA 0.5 mg L^−1^^17^. TDZ affected lingonberry shoot proliferation at low concentrations (from 0.1 to 1.0 µM) but inhibited shoot elongation^18^. However, no study has been conducted to investigate the effects of Kin and TDZ on the micropropagation of currants.

The results showed that the addition of PGRs to the nutrient medium played a key role in vegetative propagation. In the control treatment (treatment I), the percentage of regeneration was only 32%; on average, 0.36 shoots were formed from one axillary bud. Moreover, shoots were poorly formed, the average height of the formed shoots was 0.58 cm, and the number of leaves was 2.75 pieces per explant. When Kin (treatment II) was used, the regeneration rate was 64%, cultured buds produced 0.88 shoots per explant, and the shoots were isolated and tall; however, the leaves were poorly formed and the leaf blades were small. In the third treatment, the maximum shoot formation percentage was 88%. Moreover, the highest number of shoots per axillary bud (1.48 pieces) was recorded. The leaf blades of the resulting shoots were well formed. On an average, 3.68 leaves per explant were obtained, and the average shoot height was 1.23 cm.

TDZ (treatment IV) was found to be the least effective. An increase in callus tissue is observed at the base of the primary shoot. The regeneration percentage is only 56%, and 0.68 shoots regenerated from one bud. The maximum number of leaves was obtained (five leaves per explant). However, the leaves had poorly formed blades, and the average shoot height was only 0.88 cm.

**Fig. 3:**
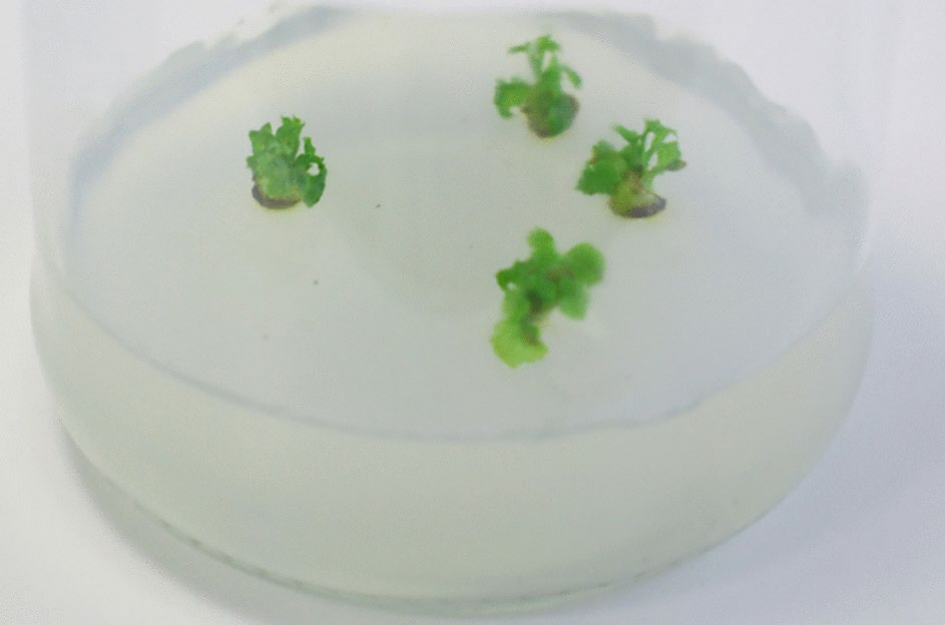
Regenerated *R. janczewskii* shoots on WPM nutrient medium with the addition of BAP 0.2 mg L^−1^ and GA 0.5 mg L^−1^.

Thus, the results showed that the use of BAP for the regeneration of the main shoot from the axillary bud of *R. janczewskii* was the most optimal treatment (Fig. 3). Therefore, the WPM nutrient medium with the addition of BAP 0.2 mg L^−1^ and GA 0.5 mg L^−1^ is an effective PGR combination for establishment in vitro culture. The resulting shoots were used to optimise the nutrient medium for micropropagation.

### Micropropagation

**Fig. 4:**
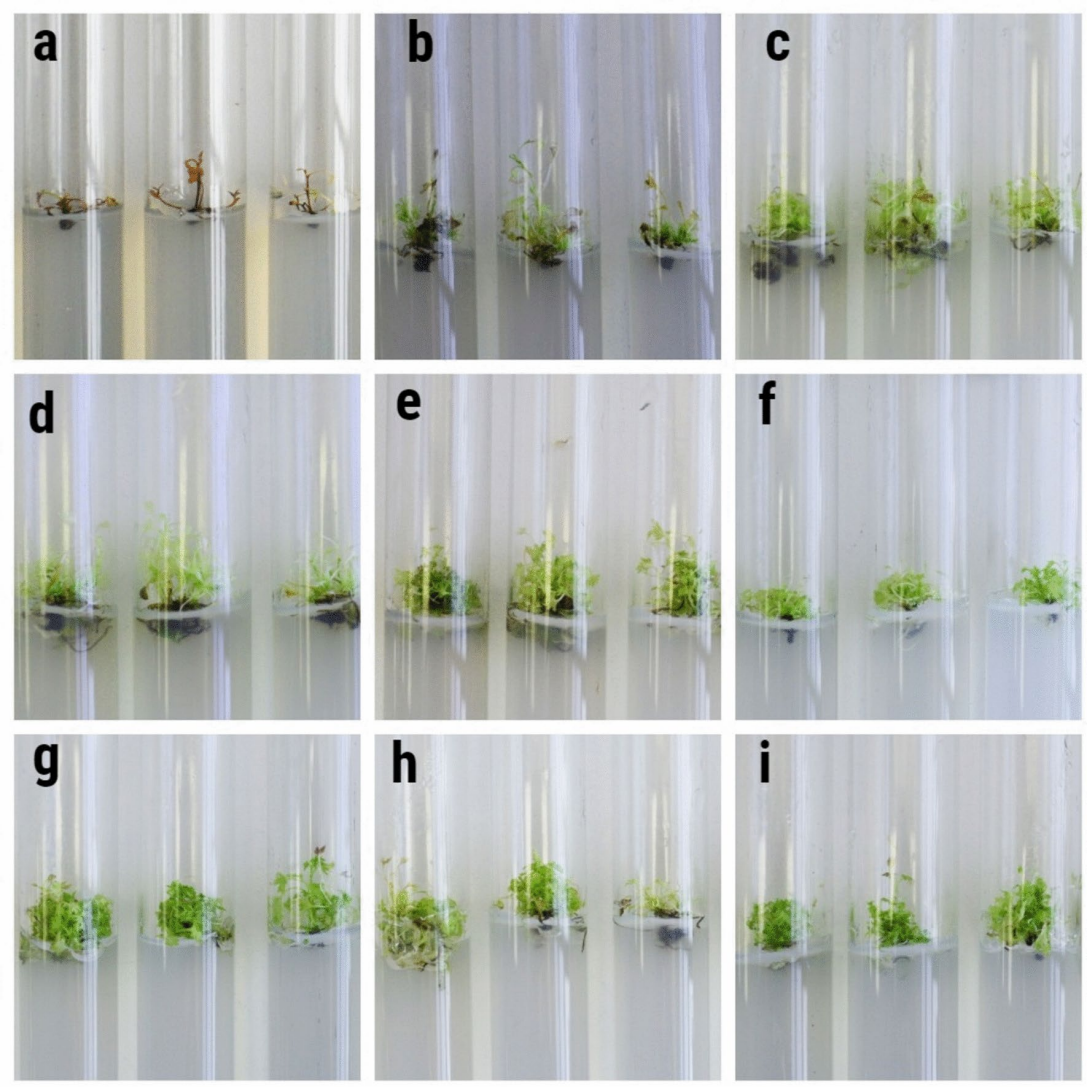
The effect of plant growth regulators (PGRs) on shoot multiplication of *R.janczewskii* in comparing the effect of three cytokinins (**a**) control; (**b**) Kin 0.25 mg L^−1^; (**c**) Kin 0.5 mg L^−1^; (**d**) Kin 0.75 mg L^−1^; (**e**) kin 1.0 mg L^−1^; (**f**) BAP 0.25 mg L^−1^; (**g**) BAP 0.5 mg L^−1^; (**h**) BAP 0.75 mg L^−1^; (**i**) BAP 1.0 mg L^−1^.

The aim of this study was to develop a micropropagation protocol for *R. janczewskii* for the first time. The effects of Kin and BAP on shoot multiplication were studied after obtaining the main shoots. The statistical analysis results indicated statistically significant differences between the treatments under consideration. WPM without hormones was used as the control. In this treatment, shoot propagation was not observed within 50 days, and explant death was observed (Fig. 4a).

The use of Kin led to the proliferation of *R. janczewskii* shoots. The number of propagated shoots depended on the concentration used. The maximum number of shoots was propagated using a concentration of 0.25 mg L^−1^. The number of shoots was 4.83 ± 0.19 pieces, the shoot height was 2.27 ± 0.05 cm and the number of leaves was 13,31 ± 0.65 pieces (Table 2, Fig. 4b). An increase in the Kin concentration led to a decrease in shoot propagation. At a concentration of 0.5 mg L^−1^, the number of shoots was 3.94 ± 0.23 pieces, while that at 0.75 mg L^−1^ was 3.57 ± 0.23 (Fig. 4c). The maximum concentration of 1.0 mg L^−1^ resulted in 3.49 ± 0.27 shoots (Fig. 4e). Importantly, the number of leaves increased from 13.31 ± 0.65 pieces to 37.20 ± 0.72 pieces per explant with an increasing Kin concentration. The use of a concentration of 0.75 mg L^−1^ led to the multiplication of 3.57 shoots (Fig. 4d). The increase in leaf growth was 20.40, while the increase in height was negligible. The propagated leaves acquired characteristic signs of deformation, exhibiting an elongated shape and narrow leaf blade. Leaf chlorosis has also been observed in some areas.

**Table 2:** Effect of plant growth regulators on shoot multiplication of *R. janczewskii.*

PGR (mg L^−1^)				50 days after culture			Accretion		
Kin	BA	GA	IBA	Number of shoots per explant	Mean shoot length (cm)	Number of leaves per explant	Number of shoots per explant	Mean shoot length (cm)	Number of leaves per explant
–	–	–	–	1.00 ± 0.00	1.41 ± 0.07	5.63 ± 0.22	0.00	− 0.05	− 0.37
0.25		0.50	0.50	4.83 ± 0.19***	2.27 ± 0.05***	13.31 ± 0.65***	3.83	0.76	8.74
0.50		0.50	0.50	3.94 ± 0.23***	2.75 ± 0.08***	16.69 ± 0.65***	2.94	1.27	11.95
0.75		0.50	0.50	3.57 ± 0.23***	1.99 ± 0.07***	25.00 ± 1.47***	2.57	0.69	20.40
1.0		0.50	0.50	3.49 ± 0.27***	3.93 ± 0.07***	37.20 ± 0.72***	2.49	2.43	32.60
	0.25	0.50	0.50	7.66 ± 0.33***	2.30 ± 0.07***	24.71 ± 0.63***	6.66	0.67	20.31
	0.50	0.50	0.50	6.14 ± 0.59***	2.81 ± 0.07***	31.23 ± 0.72***	5.14	1.08	25.63
	0.75	0.50	0.50	4.43 ± 0.21***	2.98 ± 0.15***	32.06 ± 0.38***	3.43	1.62	27.52
	1.0	0.50	0.50	3.20 ± 0.17***	4.05 ± 0.06***	32.29 ± 0.61***	2.20	2.68	27.75

***, **, *P < 0.001, 0.01, and 0.05, respectively. Less significant model terms are not shown.

The use of BAP yielded optimal results in the context of micropropagation. The highest shoot formation was observed at a BAP concentration of 0.25 and 0.5 mg L^−1^. A correlation was observed between BAP concentration and the number of shoots formed. An increase in concentration was accompanied by a decrease in the frequency of shoot propagation but led to an increase in the height and number of leaves. The maximum number of shoots (7.66 ± 0.33) was achieved with a nutrient medium supplemented with BAP 0.25 mg L^−1^, GA 0.5 mg L^−1^ and IBA 0.5 mg L^−1^. The shoot height was 2.30 ± 0.07 cm, and the number of leaves reached 24.71 ± 0.63 (Fig. 4f). The shoots formed were characterised by good structure and isolation. The leaves retained their normal structure, and the leaf blades were large. The use of WPM nutrient medium with BAP 0.5 mg L^−1^, GA 0.5 mg L^−1^ and IBA 0.5 mg L^−1^ led to the propagation of an average of 6.14 ± 0.59 shoots, with a height of 2.81 ± 0.07 cm and 31.23 ± 0.72 leaves (Fig. 4g). When using a BAP concentration of 0.75 mg L^−1^, the formation of 4.43 ± 0.21 shoots was observed (Fig. 4h). The maximum concentration of BAP (1.0 mg L^−1^) led to the propagation of only 3.20 ± 0.17 shoots, although the maximum height and number of leaves (4.05 ± 0.06 cm and 32.29 ± 0.61, respectively) was reached (Fig. 4i).

Thus, the optimal nutrient medium for micropropagation of *R. janczewskii* is BAP 0.25 mg L^−1^, GA 0.5 mg L^−1^ and IBA 0.5 mg L^−1^. A high proportion of additional shoots was obtained on this nutrient medium. Moreover, the explants retained their correct morphological structure.

### Slow-growth storage

The slow-growth storage technique is an effective approach for storing explants in in vitro culture for several months. This method enables control of plant growth and development and is cost-effective. The conditions for the slow-growth storage of *R. janczewskii* (specimen 2) shoots have not been studied. Thus, we studied the effects of various concentrations of sucrose and mannitol on the slow-growth storage of *R. janczewskii *in vitro.

**Fig. 5:**
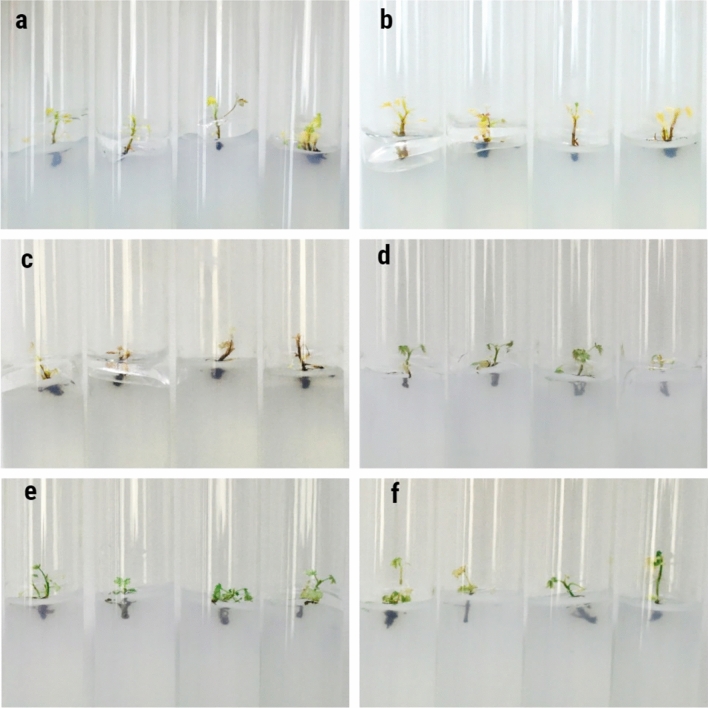
The effect of osmotic agents on slow-growth storage of *R. janczewskii* (**a**) sucrose 30 g L^−1^; (**b**) sucrose 60 g L^−1^; (**c**) sucrose 90 g L^−1^; (**d**) mannitol 10 g L^−1^; (**e**) mannitol 20 g L^−1^; (**f**) mannitol 30 g L^−1^.

A comparison of the two osmotic agents showed that mannitol had a more positive effect on the slow-growth storage of *R. janczewskii*. Sucrose yielded the weakest results. Tissue chlorosis was present in all the explants of treatment I, II, and III, and the leaves turned yellow (Fig. 5a–c). Negative dynamics in shoot height and leaf number were recorded in the III treatment of the experiment. The plant viability was low (Table 3). Only 60% of the shoots transplanted to the standard propagation medium showed repeated propagation after slow-growth storage.

**Table 3:** Growth of *R. janczewskii* microshoots during 4 months during slow-growth storage.

Osmotic agent (g L^−1^)		4 months after culture			Accretion		
Sucrose	Mannitol	Number of shoots per explant	Mean shoot length (cm)	Number of leaves per explant	Number of shoots per explant	Mean shoot length (cm)	Number of leaves per explant
30		3.0 ± 0.00	1.32 ± 0.09	8.8 ± 0.46	–	0.03 ± 0.09	0.09 ± 0.39
60		3.0 ± 0.00	1.55 ± 0.17	12.0 ± 1.41	–	0.04 ± 0.06	0.14 ± 0.59
90		3.0 ± 0.00	1.51 ± 0.09	9.4 ± 1.00	–	–0.09 ± 0.20	–0.68 ± 0.84
	10	3.0 ± 0.00	1.45 ± 0.09	8.67 ± 1.77	–	0.04 ± 0.06	0.70 ± 0.52
	20	3.0 ± 0.00	1.47 ± 0.12	7.33 ± 0.89	–	0.13 ± 0.06	1.91 ± 0.69
	30	3.27 ± 0.27	1.61 ± 0.05	10.73 ± 1.06	0.27 ± 0.27	0.00 ± 0.04	0.82 ± 0.35

The addition of mannitol had a positive effect on slow-growth storage of *R. janczewskii* microshoots. The explants retained their green colour, and leaf loss was not observed in any of the three treatments. Microshoots with 100% viability were obtained. After re-cultivation, the microshoots continued to multiply (Fig. 5d–f). The results also showed that the optimal condition for in vitro slow-growth is cultivation on a nutrient medium with the addition of mannitol 20 g L^−1^ (V treatment) (Table 3, Fig. 5e). The microshoots exhibited no chlorosis or leaf fall. The explants retained their rich green colour. The increase in shoot height (0.13 ± 0.06) and number of leaves (1.91 ± 0.69) was low, and shoot propagation was not observed. The explants remained viable for 4 months without re-cultivation.

**Fig. 6:**
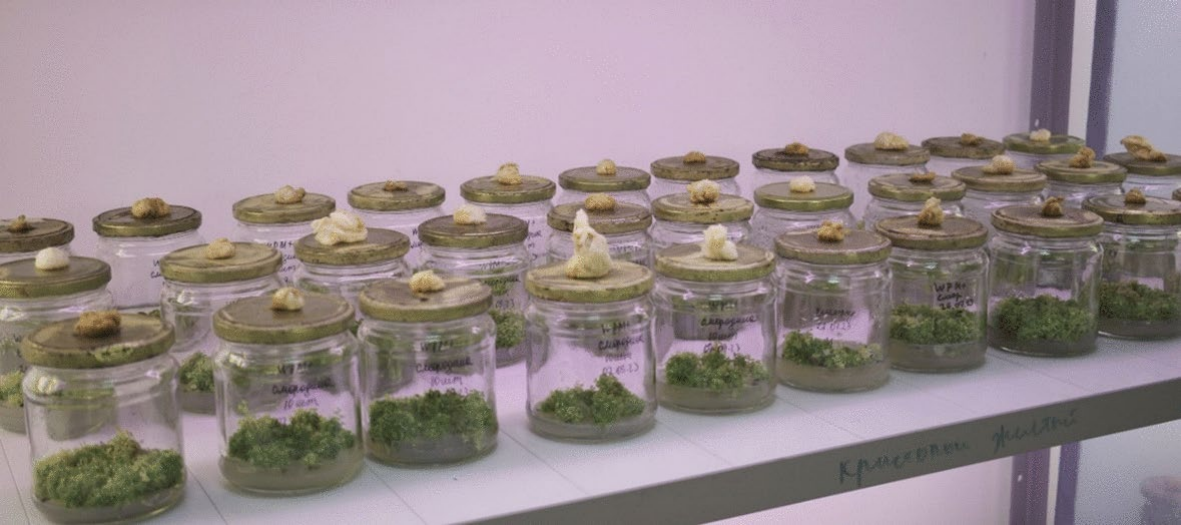
Created in vitro collection of *R. janczewskii.*

Thus, the optimal condition for in vitro slow-growth storage of *R. janczewskii* microshoots is the WPM nutrient medium with the addition of mannitol 20 g L^−1^. The explants remained viable for four months without intermediate plant transplantation. An in vitro collection of six *R. janczewskii* specimens was prepared based on an optimised protocol for micropropagation and slow-growth storage. The collection consisted of 1150 explants (Fig. 6).

## Discussion

Advances in plant biotechnology have provided new treatments for the collection, propagation, and slow-growth conservation of plant biodiversity by using in vitro culture techniques. These methods enable the efficient use of small volumes of material when working with a limited supply of plant material. Under natural conditions, *R. janczewskii* does not form large populations. Moreover, this species grows in mountainous areas, making its search even more difficult. During the expedition, we preliminarily identified six shrubs (specimens) that were presumably *R. janczewskii*. The highest point of the sample was 2351 m above sea level. To confirm the preliminarily identified specimens, molecular genetic identification was carried out. DNA barcoding was performed using the ITS2, *matK*, and *rbcL* primers to accurately determine the specimens of *R. janczewskii*. Barcodes are specific DNA segments located on chromosomes, plastids, or mitochondria that remain consistent across individuals. DNA barcoding uses molecular markers for repeated DNA-based identification, making it an effective method for identifying individuals lacking distinctive physical characteristics^19,20^. Six specimens were identified as the species in this study. The sequences of each sample using the three primers were uploaded to the international NCBI database, and the accession numbers for each specimen were obtained.

These markers were selected because of their high performance and versatility. The Consortium for the Barcode of Life has identified the *matK* + *rbcL* combination as an effective barcode for identifying flowering plants^21^. The *rbcL* marker has high versatility but low resolution, whereas *matK* offers low versatility but high resolution between different views. The combination of two markers, *matK* and *rbcL*, can help determine the maximum number of plant species. However, the combination of an internal transcribed spacer (ITS + *matK* + *rbcL*) is the most effective approach to achieve the maximum level of identification between two closely related species^22^. Thus, DNA barcoding enabled the identification of selected samples and continued research on the conservation of this rare species.

We assessed the genetic diversity of the selected samples using iPBS primers after molecular identification of *R. janczewskii*. This method is reliable for DNA fingerprinting, even in individuals new to DNA sequencing. iPBS is a cost-effective approach because it requires only basic laboratory resources for implementation, making it suitable for fingerprinting and genetic similarity assessments in plants, where it has been shown to be a robust marker system^23^.

The analysis showed a high level of polymorphism among the *R. janczewskii* specimens, ranging from 88 to 94%. This indicated a significant level of genetic diversity in this species. Our results were in agreement with those of Khapilina et al.^24^, in which the general biodiversity between populations of *A. Ledebourianum* was 67%. The mean polymorphism rates in cotton and potatoes varied from 46.2 to 100.0%^25,26^.

The identified genetic diversity has important implications for the conservation and management of biodiversity. Specimens with high levels of polymorphisms may have a greater ability to adapt to changing environmental conditions, highlighting the importance of maintaining genetic diversity for the long-term sustainability and evolution of the species. Thus, the results of the present study highlighted the importance of genetic diversity within *R. janczewskii* and its potential role in the adaptation and survival of this species under various environmental conditions.

In addition, a biochemical analysis of the fruit was performed after identifying *R. janczewskii*. The study of currant fruits is important because of their high medicinal value. For example, *Ribes nigrum* is characterised by high antioxidant activity due to its high content of biologically active compounds such as polyphenols, which include phenolic acids, flavonoids, and anthocyanins, as well as vitamins, mainly vitamin C^27,28^. However, no work on the biochemical composition of *R. janczewskii* has been previously published. Thus, the fruits of six *R. janczewskii* specimens were examined in the present study. The analysis showed that fruits are a valuable source of nutrients. There were no statistically significant differences in the content of biologically active substances among the six specimens. These results highlighted the importance of *R. janczewskii* as a valuable source of vitamins and other biologically active compounds that promote health and strengthen the immune response. In this regard, the use of the rare species *R. janczewskii* in crossing to obtain cultivated varieties of black currant is an urgent task.

Micropropagation is a widely accepted method of conserving rare and valuable species^29^. The first step in optimising the protocol was to select a sterilising agent. Hydrogen peroxide is a safe and effective sterilisation agent. Hydrogen peroxide is used as a steriliser for fruit crops at concentrations ranging from 3 to 30%^30^. *Prunus persica* explants were sterilised at concentrations of 10% and 20%^31^. For *R. janczewskii*, the optimal concentration was a 12% hydrogen peroxide solution. This mode neutralises microflora without damaging plant tissues.

Successful regeneration of the main shoot from an axillary bud and micropropagation of *R. janczewskii* was achieved by studying the composition of plant growth regulators. BAP and Kin are widely used antioxidants. For example, a high propagation coefficient was noted in black currant at a concentration of 2.5 mg L^−1^, where up to 7.6 new shoots were propagated^30^. More shoots were obtained from *Polygala myrtifolia* L. on medium containing Kin than BAP^32^. According to the results of our study, positive results for all phenological parameters were obtained using the BAP. The maximum number of shoots was obtained when using a concentration of 0.25 mg L^−1^.

In addition, BAP is effective for *Ribes aureum*. A previous study indicated that a concentration of 5 μM is optimal for the propagation of additional shoots in in vitro culture^33^. We found that BAP 0.2 mg L^−1^ and GA 0.5 mg L^−1^ was an effective PGR combination for in vitro culture establishment, while BAP 0.25 mg L^−1^, GA 0.5 mg L^−1^ and IBA 0.5 mg L^−1^ resulted in the propagation of additional shoots. Similar results to ours were obtained during the propagation of *Ribes magellanicum*, where the highest propagation was obtained when using BAP at concentrations of 0.25 or 0.50 mg L^−1^^34^. Increasing the BAP concentration to 0.75 and 1.0 mg L^−1^ increased the shoot height to 2.98 cm and 4.05 cm, respectively. The maximum number of leaves relative to the remaining BAP concentration was recorded. High BAP content leads to shoot elongation in black currants^35^.

The micropropagation method has the additional advantage of enabling the long-term conservation of plant material in in vitro culture^36^. This method is used for a wide range of plant species, and protocols are often optimised for the object of study^37^. In general practice, the osmotic agents sucrose and mannitol are widely used for slow-growth storage^38^. They serve as both carbon sources and osmotic stressors as they tend to reduce water potential and limit water availability to explants. This prevents cell elongation and division, limits cell growth and prolongs cell storage. Mannitol is used as an osmotic stressor to maintain slow growth in many plant species^39,40^ and increase the re-cultivation interval^41^. In the present study, the effects of sucrose were unclear. There was chlorosis of the explants and sometimes death. In contrast to our findings, other plant species have shown positive results. For example, *Castanea sativa* was stored for 48 months with 30 g L^−1^ sucrose, *Citrus jambhiri* remained viable for a year on WPM with 25 g L^−1^ sucrose, and *Prunus avium* × *P. cerasus* explants was stored for 16 months in the dark with 60 g L^−1^ sucrose^42–44^.

However, during slow-growth storage of *R. janczewskii*, mannitol had a positive effect. The beneficial impacts of mannitol have been validated in black currants as well. The explants were stored in nutrient medium supplemented with 2% sucrose and 2% mannitol for 18 months. Mannitol has a beneficial effect on plum shoots, where explants are stored for up to 30 months^45^. *Vitis vinifera* was stored for 12 months, the mannitol content increased to 2.5%^46^. The addition of 10 g L^−1^ mannitol enabled the preservation of *Vitis heyneana* explants for 12 months^47^.

## Methods

**Fig. 7:**
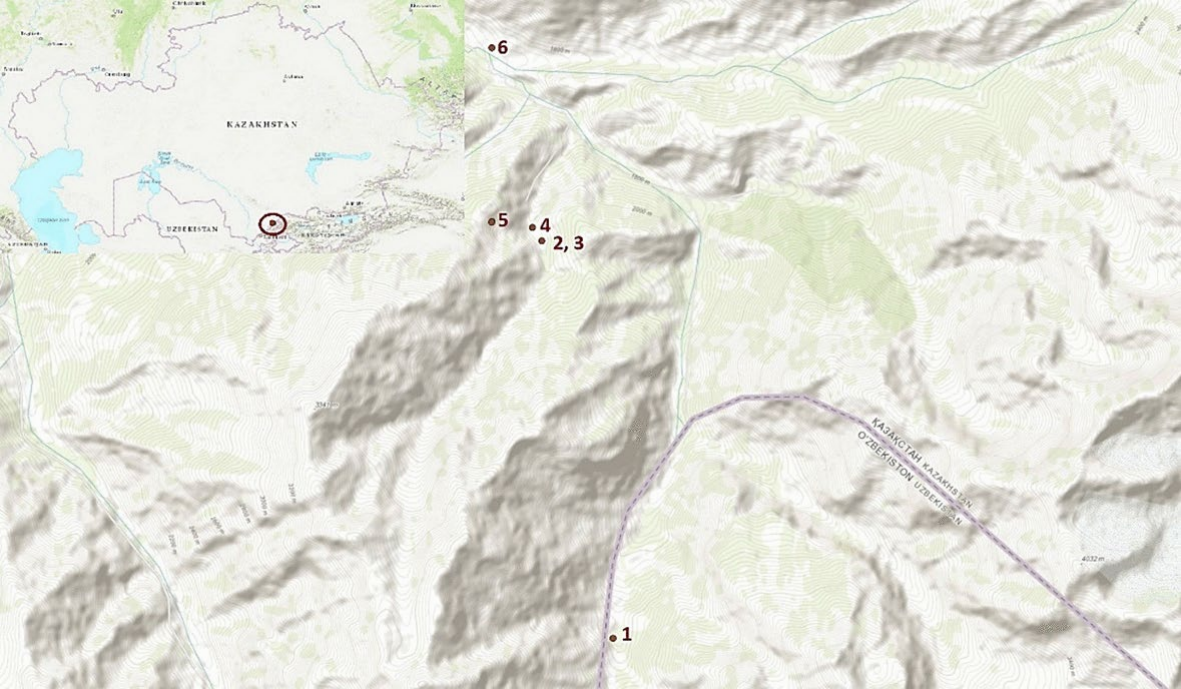
Locations of sample collection of *R. janczewskii* in the Kazakhstan Sayram-Ugam National Park territory.

The plant material of *R. janczewskii* specimens was used as a research subject. Material was collected from the Turkestan region, Tolebi District in the Sayram-Ugam State National Natural Park. Six wild specimens were identified during the expedition. The *R. janczewskii* specimens were preliminarily identified by Alima Umirzakova, a member of the scientific staff of the Sairam-Ugam State National Nature Park. The coordinates and absolute altitude of each specimen were determined using a Garmin GPS MAP 60 CX Navigator (Olathe, KS, USA). The longest distance between Specimens (1 and 6) was 2965 m. The shortest distance (specimens 4 and 5) was 11,720 m (Fig. 7). The GPS data of *R. janczewskii* GPS specimens are as follows: 1, 70°23.994′ E and 42°06.526′ N; 2, 70°23.422′ E and 42°08.891′ N; 3, 70°23.347′ E and 42°08.971′ N; 4, 70°23.433′ E and 42°08.883′ N; 5, 70°23.020′ E and 42°09.002′ N; and 6, 70°23.021′ E and 42°10.040′ N. Height above mean sea level ranged from 1711 to 2351 m. The collected wild specimens of *R. janczewskii* in Sairam-Ugam State National Nature Park were deposited for storage to the Astana Botanical Garden (AGB) under identification numbers NUR006890–NUR006895 in the form of a herbarium. The herbarium was prepared by Anna Muranets. Axillary buds for micropropagation were stored at 4 °C until use. Fresh leaves for molecular analysis were stored at − 20 °C until use.

### DNA barcoding

DNA barcoding was used for species identification of the selected specimens. Leaves of *Ribes janczewskii* were sampled from each specimen. DNA was extracted using the modified CTAB method (2% CTAB, 1.4 M NaCI, 0.2% 2-mercaptoethanol, 20 mM EDTA, 100 mM Tris–HCl, pH 8.0)^48^. The quality and quantity of extracted DNA were verified using an ultraviolet spectrophotometer (NanoDrop1000; Thermo Scientific, Wilmington, NC, USA), and each sample was diluted to 50 ng μL^−1^ in sterile distilled water.

A PCR reaction mixture of 20 µL comprised the following: 3 µL of template DNA (50 ng), 2 µL PCR buffer (10 ×), 1 µL MgCl_2_ (25 mM), 1,5 µL of both forward and reverse primers (10 pmol) (Table 4), 0.25 µL Dream Taq polymerase, 1 µL dNTPs (10 mM) and deionised distilled water. The primers were synthesised at the Laboratory for the Development of Molecular Diagnostic Approaches at the National Center for Biotechnology. The PCR cycling conditions were as follows: 95 °C for 5 min for DNA denaturation; 35 cycles of 95 °C for 1 min, 52–58 °C for 1 min, and 72 °C for 1 min; and 72 °C for 10 min. The amplified PCR products were electrophoresed on a 1% agarose gel (1 × TBE buffer & 0.5 μg/mL ethidium bromide) to check for the presence or absence of bands. DNA digested with a 1000 bp DNA Ladder (Fermentas, Burlington, Canada) was used as the DNA marker, and bands were visualised under UV light and photographed using a PharosFX Plus Imaging System (Bio-Rad Laboratories Inc., Hercules, CA, USA). PCR was performed at least twice to ensure the reproducibility of the results.

**Table 4:** Primers used for amplification of *matk, rbcl* and ITS2 genome regions.

Gene Region	Name	Sequence	Optimal annealing T, °C
*matK*	3F_KIMf	5′-CGTACAGTACTTTTGTGTTTACGAG-3′	52
	1R_KIMr	5′-ACCCCATTCATCTGGAAATCTTGGTTC-3′	
*rbcL*	rbcLa_F	5′-ATGTCACCAACAAACAGAGACTAAAGC-3′	58
	rbcLa_R	5′-GTAAAATCAAGTCCACCRCG-3′	
ITS2	ITS4	5′-TCCTCCGCTTATTGATATGC-3′	55
	ITSS	5′-GGAAGTAAAAGTCGTAACAAG-3′	

Purification of the PCR products was performed using shrimp alkaline phosphatase (SAP) and ExoI (Thermo Scientific) enzymes. The reaction mixture (20 μL) contained 1 × SAP buffer, 10 μL of PCR product, 3 units of exonuclease ExoI, and 1 unit of SAP and was incubated at 37 °C for 30 min, followed by inactivation of the enzyme at 75 °C for 15 min. The initial incubation hydrolysed excess primers and dephosphorylated the nucleotides, and the second incubation inactivated the enzymes, minimising the loss of the PCR product so that further sequencing could be performed without further purification using the columns.

DNA sequencing was performed using an ABI Prism BigDye Terminator reagent kit version 3.1 (Austin, TX, USA), followed by analysis of the reaction products on an automated sequencer (Applied Biosystems 3730 DNA Analyser, Tokyo, Japan). The nucleotide sequences of the analysed samples were retrieved and edited using SeqMan^49^.

### Assessment of genetic diversity

We used DNA profiling methodologies centred on conserved sequences of interspersed repeats to evaluate genetic variability within wild specimens of *R. janczewskii*. Specifically, we used conserved sequences associated with tRNA primer-binding sites (iPBS) of long-terminal repeat retrotransposons as primers for PCR amplification. The genetic variability of the endemic *R. janczewskii* specimens was analysed using iPBS primers designed by Kalendar et al.^50^.

The PCR reaction mixtures (25 µL) comprised the following: DNA 40 ng, 2,5 μL PCR buffer (10 ×), 2,5 μL dNTPs (2,5 mM), 2,5 μL iPBS primer (10 pmol), 0,2 μL Dream Tag polymerase (5 U/μL) and deionised distilled water. The PCR cycling conditions were as follows: 95 °C for 2 min for DNA denaturation; 35 cycles of 95 °C for 20 s, 52–58 °C for 30 s, and 72 °C for 1 min; and 72 °C for 2 min. The primers used and annealing temperatures are listed in Table 5. PCR products were separated by electrophoresis at 70 V for 10 h on a 1.2% agarose gel with 1 × TBE buffer and ethidium bromide. The Thermo Scientific GeneRuler DNA Ladder Mix (100–10,000 bp) was used as the standard DNA ladder. PCR products were visualised using a PharosFX Plus Imaging System (Bio-Rad Laboratories Inc.). All PCR and electrophoresis analyses were repeated three times and only clear sharp bands were scored. PCR fragments were scored as present (1) or absent (0) to construct the binary matrix.

**Table 5:** Data of PBS primers used in this study.

№	Sequence ID	Sequence	Optimal annealing, Ta (°C)
1	2228	CATTGGCTCTTGATACCA	54.0
2	2240	AACCTGGCTCAGATGCCA	55.0
3	2395	TCCCCAGCGGAGTCGCCA	52.8
4	2222	ACTTGGATGCCGATACCA	53.0
5	2224	ATCCTGGCAATGGAACCA	55.4
6	2229	CGACCTGTTCTGATACCA	52.5
7	2230	TCTAGGCGTCTGATACCA	52.9
8	2232	AGAGAGGCTCGGATACCA	55.4
9	2237	CCCCTACCTGGCGTGCCA	55.0
10	2270	ACCTGGCGTGCCA	65.0
11	2393	TACGGTACGCCA	51.0
12	2394	GAGCCTAGGCCA	56.5
13	2395	TCCCCAGCGGAGTCGCCA	52.8
14	2398	GAACCCTTGCCGATACCA	51.0

### Biochemical composition of berries

**Fig. 8:**
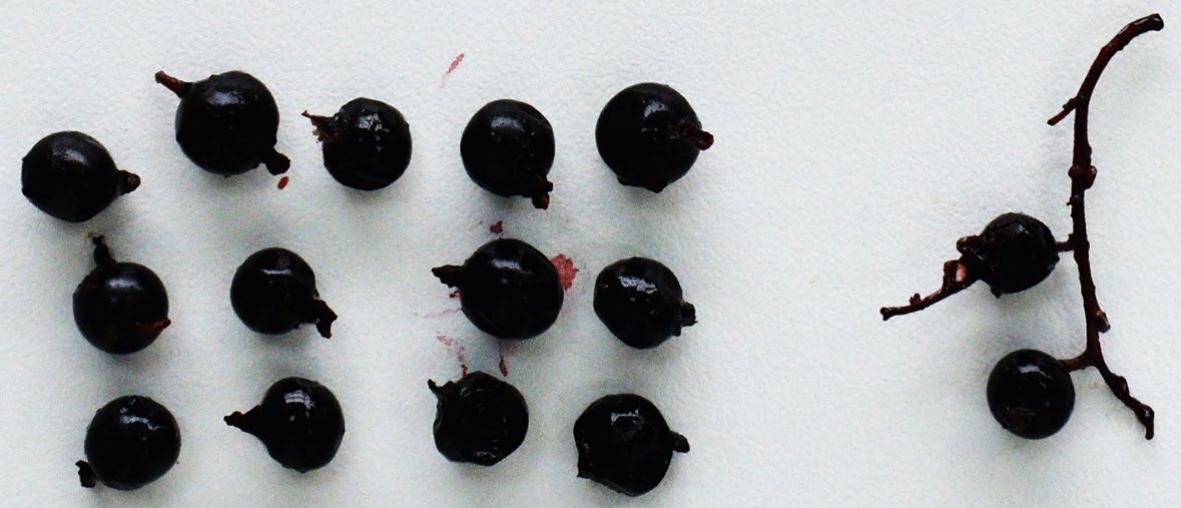
Berries of *Ribes janczewskii.*

Mature berries were harvested at the beginning of September and were frozen at − 20 °C. Berries were harvested from six shrubs for the analysis of vitamins C, E, B5, and quercetin content (Fig. 8). To ensure the fruits were at optimal ripeness, we assessed ripeness visually and by touch, selecting only those fruits that exhibited the characteristic color, texture, and firmness of fully ripened specimens. The timing of harvest was consistent across the sample population, ensuring that all fruits were at a similar stage of maturity. For each analysis, 100 g of fruit was used. High-performance liquid chromatography (HPLC) (vitamins C, E, and B5) and spectrophotometry (quercetin) were used to determine the biologically active substances. The data presented are the means of measurements taken from these six shrubs, with each measurement repeated three times to account for technical variability.

The vitamin C content was determined using a method based on the extraction of vitamin C from the sample with a solution of metaphosphoric acid, followed by the reduction of L(+)-dehydroascorbic acid to L(+)-ascorbic acid, and determination of the total content of L(+)-ascorbic acid using highly effective liquid chromatography with spectrophotometric detection at a wavelength of 265 nm. The l-ascorbic acid peak was identified by comparing the retention time in the chromatograms of the sample and calibration solutions.

The vitamin E content was determined using high-performance liquid chromatography. The method was based on the determination of α, β, γ and δ-tocopherols in a sample solution by high-performance liquid chromatography with photometric detection. The sample material was saponified using a potassium hydroxide solution to prepare the sample solution, followed by analyte extraction. The compounds to be determined were identified based on their retention times and quantified using an external standard based on the peak area or peak height measurements.

The vitamin B5 content was determined using a method based on the acid hydrolysis of pantothenic acid to form pantolactone, extraction of the latter with chloroform, and determination of its content in solution using liquid chromatography. The mass fractions of the free and bound pantothenic acids were analysed. Quantitative determination of the flavone derivative quercetin was performed to determine the flavonoid content. The analysis of the reaction products in a solution of aluminium chloride was conducted using spectrophotometry at a wavelength of 415 nm.

### Sterilisation and establishment of in vitro culture

The axillary buds of the annual shoots of wild specimens of *R. janczewskii* (specimen 2) were used to establish in vitro cultures. The effectiveness of hydrogen peroxide solutions (H_2_O_2_) was studied for the sterilisation of explants was studied. The main sterilisation procedure was performed under sterile conditions. Three sterilisation treatments were studied to select the optimal concentration: I, 6% H_2_O_2_; II, 12% H_2_O_2_; III, 24% H_2_O_2_; and an exposure time of 5 min. Next, the explants were thoroughly washed with sterile distilled water and dried on filter paper. The explants were cultured in hormone-free woody plant medium (WPM) to assess the effectiveness of sterilisation. Fifteen explants were planted in each treatment.

### Regeneration of the main shoot

After obtaining sterile and viable explants, various hormones were tested, including kinetin (Kin), 6-benzylaminopurine (BAP), thidiazuron (TDZ) and gibberellic acid (GA). For this purpose, the WPM nutrient medium was used. The following treatments were explored: I, WPM without plant growth regulators (PGRs); II, Kin 0.2 mg L^−1^ and GA 0.5 mg L^−1^; III, BAP 0.2 mg L^−1^ and GA 0.5 mg L^−1^; and IV, TDZ 0.2 mg L^−1^ and GA 0.5 mg L^−1^. For each study treatment, 25 explants were cultured and observed for 21 days. The explants were grown in culture vessels in a green room during all stages of the study. LED strips (SMD 5050) were installed on each shelf in the green room at 60 led/m. The photoperiod was 16/8, and the temperature was maintained at 24–26 °C.

### Micropropagation

A micropropagation study was conducted after obtaining the main microshoots. The effect of plant growth regulators was studied: Kin and BAP (0.25, 0.5, 0.75 and 1.0 mg L^−1^), GA (0.5 mg L^−1^) and indole-3-butyric acid (IBA) (0.1 mg L^−1^)*.* The following parameters were examined to study the effectiveness of these conditions: shoot height, number of shoots, and number of leaves. For each study treatment, 35 explants were cultured and observed for 50 days.

### Slow-growth storage

The effect of increased concentrations of sucrose and mannitol on the in vitro slow-growth storage of *R. janczewskii* (specimen 2) was studied. The following treatments based on the nutrient medium were studied WPM: I, sucrose 30 g L^−1^; II, sucrose 60 g L^−1^; III, sucrose 90 g L^−1^; IV, mannitol 10 g L^−1^; V, mannitol 20 g L^−1^; and VI, mannitol 30 g L^−1^. Explants were grown in culture vessels in a green room. Shoot height and the number of leaves were used to determine the effectiveness of these conditions. Fifty explants of each study treatment were cultured. Each explant consisted of three shoots because single shoots had a low survival rate during the early stages of the study. Data were collected in the 4th month of cultivation.

### Statistical analyses

The experimental results were subjected to ANOVA, and significant differences were selected using Tukey’s post-hoc test using SPSS (version 25.0 (IBM Inc., New York, NY, USA). The data are expressed as means ± standard error of three independent experiments.

## Conclusion

The study and conservation of rare and endemic species are important for maintaining biodiversity. This study demonstrates the importance of determining the optimum conditions for micropropagation and slow-growth storage. The optimised protocol enables the conservation and propagation of *R. janczewskii*. Additionally, identification of the plant at the molecular level was achieved through DNA barcode analysis using three distinct DNA barcodes published in the NCBI. Intraspecific polymorphisms with high genetic diversity were also studied. This indicates a high ability to adapt to unfavourable environmental conditions. For the first time, the biochemical composition of the fruit was studied, which showed a high content of biologically active substances. Moreover, despite the genetic diversity, there were no differences in the content of biologically active substances between the specimens. As a result of our study, an in vitro collection of *R. janczewskii* consisting of 1150 explants was created.

## Supplementary Information

Supplementary Information.

## Data Availability

The datasets analysed during the current study are available in the National Center for Biotechnology Information (NCBI) repository. PP702917: https://www.ncbi.nlm.nih.gov/nuccore/PP702917.1 PP464114: https://www.ncbi.nlm.nih.gov/nuccore/PP464114.1 PP464115: https://www.ncbi.nlm.nih.gov/nuccore/PP464115 PP709330: https://www.ncbi.nlm.nih.gov/nuccore/PP709330 PP708896: https://www.ncbi.nlm.nih.gov/nuccore/PP708896 PP708897: https://www.ncbi.nlm.nih.gov/nuccore/PP708897 PP708898: https://www.ncbi.nlm.nih.gov/nuccore/PP708898 PP708899: https://www.ncbi.nlm.nih.gov/nuccore/PP708899 PP708900: https://www.ncbi.nlm.nih.gov/nuccore/PP708900 PP708901: https://www.ncbi.nlm.nih.gov/nuccore/PP708901 PP493202: https://www.ncbi.nlm.nih.gov/nuccore/PP493202 PP493204: https://www.ncbi.nlm.nih.gov/nuccore/PP493204 PP493205: https://www.ncbi.nlm.nih.gov/nuccore/PP493205 PP493206: https://www.ncbi.nlm.nih.gov/nuccore/PP493206 PP493207: https://www.ncbi.nlm.nih.gov/nuccore/PP493207 PP708902: https://www.ncbi.nlm.nih.gov/nuccore/PP708902.
