# Supplementary material for: In Vitro conservation and genetic diversity analysis of rare species Ribes janczewskii
Source: Sci Rep. 2024 Dec 28;14:31117. doi: 10.1038/s41598-024-82320-y (PMC11680832; doi:10.1038/s41598-024-82320-y)

Supplementary Information for Figure 1

Electrophoretic pattern of *R. janczewskii* fragments using iPBS primer

2240 **2224** **2229**

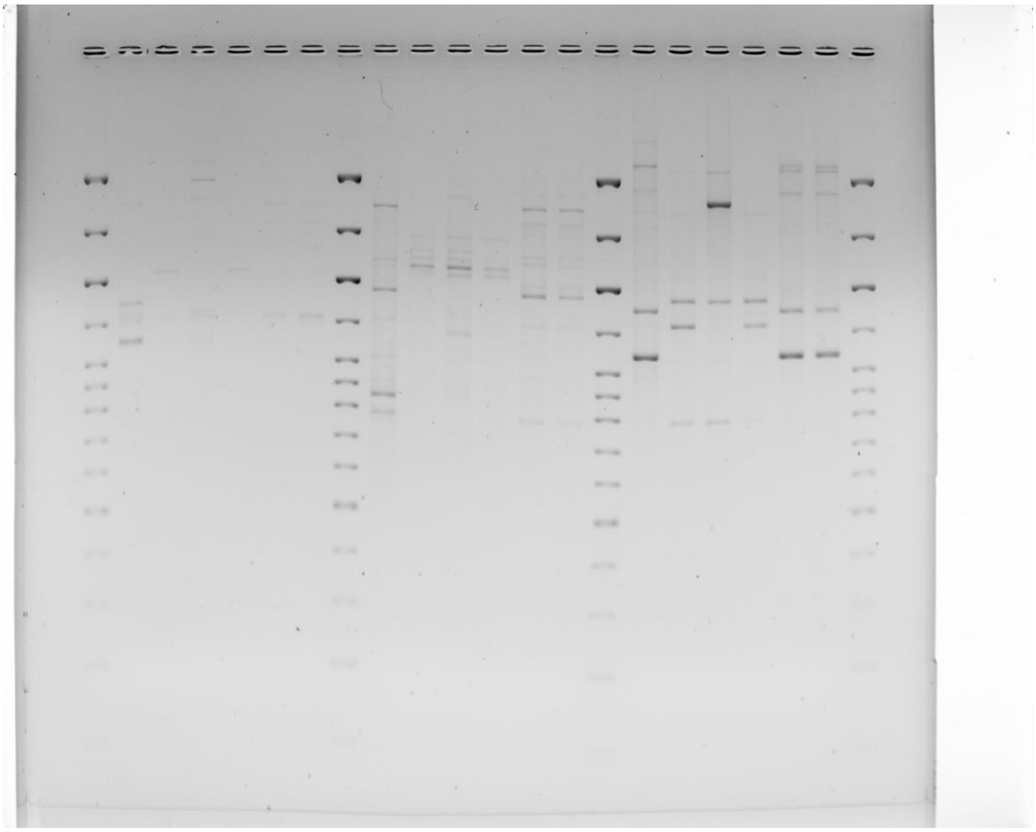

2393 **2228**

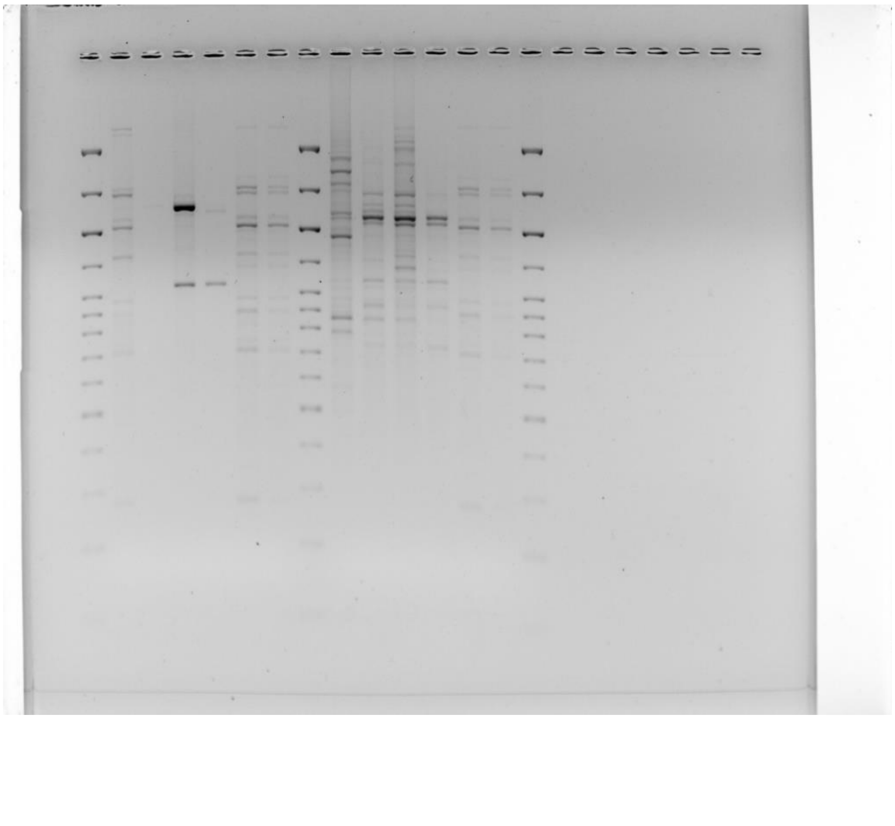

Supplement: Supplementary file 1 — Supplementary Information. [file 41598_2024_82320_MOESM1_ESM.pdf]
